# Investigation of Glandular Trichome Proteins in *Artemisia annua* L. Using Comparative Proteomics

**DOI:** 10.1371/journal.pone.0041822

**Published:** 2012-08-08

**Authors:** Ting Wu, Yejun Wang, Dianjing Guo

**Affiliations:** School of Life Sciences and State Kay Laboratory of Agrobiotechnology, The Chinese University of Hong Kong, Shatin, Hong Kong SAR, People's Republic of China; Lawrence Berkeley National Laboratory, United States of America

## Abstract

Glandular secreting trichomes (GSTs) are called biofactories because they are active in synthesizing, storing and secreting various types of plant secondary metabolites. As the most effective drug against malaria, artemisinin, a sesquiterpene lactone is derived from GSTs of *Artemisia annua*. However, low artemisinin content (0.001%∼1.54% of dry weight) has hindered its wide application. We investigate the GST-expressed proteins in *Artemisia annua* using a comparative proteomics approach, aiming for a better understanding of the trichome proteome and arteminisin metabolism. 2D-electrophoresis was employed to compare the protein profiles of GSTs and leaves. More than 700 spots were resolved for GSTs, of which ∼93 non-redundant proteins were confidently identified by searching NCBI and Artemisia EST databases. Over 70% of these proteins were highly expressed in GTSs. Functional classification of these GSTs enriched proteins revealed that many of them participate in major plant metabolic processes such as electron transport, transcription and translation.

## Introduction

Trichomes are hair cells or leaf hairs originated from the outgrowth of specialized epidermal cells on leaf or other organs of plants. They play an important role in plant taxonomy due to its variance in size, shape, morphology, cell number and composition [1]. Normally, trichomes are divided into two general types: non-glandular trichomes (also known as simple trichomes) and glandular trichomes (also called glandular secreting trichomes, GSTs). These two types of trichomes are proposed to serve different functions. Generally, non-glandular trichomes function in water absorption, seed dispersal, deterring herbivores and so on, whereas GSTs are major sites for biosynthesis, storage and secretion of diverse classes of plant secondary metabolites. These trichome-specific phytochemicals are actively involved in host defense and pest attraction in many plant species [2]. Most of these compounds (e.g. essential oils, flavonoids, phenolics and terpenoids) also have high commercial values in pharmaceutical, food and cosmetic industries. Artemisinin, a sesquiterpene lactone isolated from GSTs of *Artemisia annua* L., is currently regarded as the most effective drug against malaria [3], [4]. However, the low concentration of artemisinin in *A. annua* (ranging from 0.01%∼1.1% of the dry weight) is one of the major factors that hinders the wide application of this important drug. In order to increase the artemisinin yield, tremendous efforts have therefore been dedicated to elucidate the function of GSTs and the artemisinin biosynthetic pathway. With gas chromatography-tandem mass spectrometry, high performance liquid mass spectrometry or nuclear magnetic resonance, many intermediate compounds participating in artemisinin production have been identified [5]. Several gene coding enzymes involved in the artemisinin biosynthesis pathway (e.g. P450, DBR2) have been cloned and functionally characterized [6]–[10]. Moreover, recent global surveys of GST transcriptomes in *A. annua* were conducted using high throughput pyrosequencing technology [11], [12]. Such efforts have provided a useful EST resource for gene discovery. However, abundance of expressed transcripts may not represent the protein activity and proteomic studies of *A. annua* GSTs are necessary for further elucidating the cellular machinery operating in GSTs. We therefore conducted a comparative proteome study aiming to unravel candidate proteins that participate in artemisinin biosynthesis and transport, as well as in GST development. We isolated GSTs from 4-month old *A. annua* leaves for the proteome profiling, and 2DE gel electrophoresis and matrix assisted laser desorption/ionization time-of-flight mass spectrometry (MALDI-TOF/TOF MS) were employed for separation and analysis of the total proteins. Differentially expressed proteins between GSTs and the corresponding leaves were investigated. Over 700 spots excised from 2DE gel of GSTs were subjected to MS analysis, of which ∼93 non-redundant proteins were confidently identified by searching NCBI and Artemisia EST databases. We found that over 70% of these proteins were highly expressed in GTSs compared to leaves. Functional classification revealed that most of them participate in the electron transport chain, metabolism, transcription/translation, etc. Some of these candidates may provide potential targets for genetic manipulation of GSTs for enhanced artemisinin production in *A.annua*.

## Materials and Methods

### Materials

The seeds of *A. annua* were purchased from You Yang, Si Chuan Province, China. The seeds were germinated under controlled conditions (average 30°C) in the greenhouse of the School of Life Sciences, Hong Kong Baptist University. GST samples were collected from the 4^th^ leaves counting from the bottom of 16–18 week-old plants. Leaf samples were harvested from the same plants before mechanical removal of trichomes for comparison. Three independent groups of plant materials were included in the study.

### Isolation of Glandular Trichomes

Glandular trichomes were isolated from *A. annua* leaves as described previously [7]. The isolated GSTs were then subjected to additional sucrose gradient purification. Leaves were imbibed with ice-cold distilled water for 1 hr. Subsequently, 20–30 g of fresh-weight leaves were placed into a 350 ml bead beater chamber (BioSpec Products, Inc., Bartlesville, OK, USA). The chamber was then filled with 80–100 g of glass beads (0.5 mm diameter, BioSpec Products, Inc., Bartlesville, OK, USA), XAD-4 resin (1 g/g plant material) (Amberlite® XAD® 4, SUPELCO, Sigma-Aldrich Chemical Co., Bellefonte, PA, USA), and isolation buffer (25 mM MOPSO, pH 6.6, 200 mM sorbitol, 10 mM sucrose, 5 mM thiourea, 2 mM dithiothreitol, 5 mM MgCl_2_, 0.5 mM sodium phosphate, 0.6% (w/v) methylcellulose and 1% (w/v) polyvinylpyrrolidone (PVP) (Mr 40000). The trichomes were gently abraded from the leaves on ice for 3×1 min with 1 min intervals. After abrasion, the crude cellular extract was separated by sequentially filtering through a 300 µm and a 105 µm nylon mesh (Small Parts Inc., Miami Lake, FL, USA). The residual plant materials were also rinsed twice with rinse buffer (Isolation buffer without PVP and methylcellulose) and filtered. The resulting solution of mixed cells was then concentrated by centrifugation (10 min, 4°C, 6000 rcf) using an eppendorf 5810R centrifuge (Eppendorf, Germany) and was resuspended in 15–20 ml pre-cold rinse buffer. 3–4 ml of the concentrated mixture was gentled layered on the top of 30 ml 40% sucrose solution along the wall of 50 ml centrifuge tube (Neptube, Germany). The tube was then put into the swing-bucket rotor and was centrifuged at 4°C for 10 min at 750 rcf (with acceleration levels 5 and deceleration level 0). Consequently, the upper layer was carefully transferred to the new tubes to be concentrated. After 3 washes with ice-cold distilled water, the isolated trichomes were ready for protein extraction and microscopic analysis. Leaf samples and trichomes were all frozen in liquid nitrogen and stored at −80°C.

### Protein Extraction

The extraction procedure was performed as described previously [13], [14] with the following minor modification. The frozen plant material was ground into fine powder with a mortar under liquid nitrogen. The obtained powder was then incubated with 1∶10 (w/v) precipitation solution containing 10% w/v trichloroacetic acid and 0.07% w/v 2-mercaptoethanol in ice-cold acetone and was kept overnight at −20°C. After centrifugation at 14000 g for 30 min at 4°C, the protein pellets were washed 2∼3 times with ice-cold acetone containing 0.07% w/v 2-mercaptoethanol and freeze-dried. Dried samples were re-suspended in lysis buffer (7 M urea, 2 M thiourea, 4% 3-(3-cholamidopropyl) dimethylammonio-1-propane sulfonate, 100 mM dithiothreitol and 2% Bio-Lyte Carrier Ampholyte solution pH 3–10) and incubated at room temperature for 2 hrs with 10 min sonication per hour. The protein lysate was clarified by centrifugation through 0.45 µm spin filter units (Ultrafree-MC, Millipore, USA). Protein concentration was measured using a quick start bradford dye reagent (Bio-Rad, USA) using BSA (4 mg.ml^−1^) as standard.

### Two-Dimensional Gel Electrophoresis

900 µg of protein in lysis buffer containing a trace amount of bromophenol blue were used to rehydrate immobilized pH gradient (IPG) strips of 18 cm with a linear pH gradient of 4–7 (Readystrip, Bio-Rad, USA). The solution was then incubated for 20 hrs at room temperature. Isoelectric focusing was carried out on an Ettan IPGphor unit (GE Healthcare, USA) using the following settings: 100 V for 3 h, 300 V for 3 h, 1000 V for 1 h, 3000 V for 24000 Vhr and 8000 V for 64000 Vhr at 20°C with a maximum current setting of 50 mA/strip. After the isoelectric focusing, the IPG strips were first equilibrated for 10 min in equilibration buffer (50 mM Tris, pH 8.8, 6 M urea, 20% v/v glycerol, 2% w/v sodium dodecyl sulfate) containing 2% w/v dithiothreitol, and equilibrated for another 10 min with the same buffer containing 2.5% w/v iodoacetamide. After equilibration, the IPG strips were placed on the top of 14% polyacrylamide gel and subjected to electrophoresis in a vertical PROTEAN**®** II xi Cell electrophoresis system (Bio-Rad, USA) at 80 V for 1 hr and 200 V for approximate 7 hrs. After fixation for 1 hr with a fixation buffer (40% menthol, 10% acetic acid), the gels were stained overnight with “Blue silver” staining solution (10% phosphoric acid, 10% ammonium sulfate, 20% anhydrous methanol, 0.12% Brilliant Blue G-250). Finally, the gels were distained using 500 ml Milli-Q water for 30 min and this step was repeated 3 times. After staining and distaining, the gels were scanned under visible light at 300 dpi using ImageScanner (Amershan Biosciences) with Labscan software. Analysis was carried out using the computer program, ImageMaster 2D Platinum 5.0 (Amershan Biosciences). Spots were detected automatically and manually edited and deleted to remove technical artifacts. For alignment and matching of spots, one gel was chosen as a reference and two spots were manually selected as landmarks. The volume of each spot was normalized against total spot volume and the percentage of resulting spot volume was used for comparison. Only spots with >2 fold changes and P values<0.05, or those appearing only on three gels of glandular trichomes were considered as significant up-regulated ones.

### In Gel Digestion and Protein Identification by Mass Spectrometry

Spots of interest were excised from the two-dimensional electrophoresis (2-DE) gel obtained from trichomes after image analysis. The spots were transferred to 1.5 ml microcentrifuge tubes to be destained with 50 mM ammonium bicarbonate, dehydrated with 99.9% HPLC grade acetonitrile and then vacuum dried completely by a SpeedVac (LABCONCO). For the rehydration and digestion of proteins, 5–7 µl 25 mM NH_4_HCO_3_ containing 40 ng/µl sequence grade modified trypsin (Promega, Madison, USA) were added to each sample and samples were incubated for 30 minutes on ice. In-gel digestion with trypsin was continued over night at 37°C. After sonication for 10 min, the supernatant was removed to a new tube, and the reaction was stopped by adding 10 µl of extraction buffer (50%, 2.5% trifluoracetic acid), followed by sonication for another 10 min. The extracts were combined and completely dried under vacuum and dissolved in 5 µl 0.1% trifluoracetic acid. Digested sample solution (1.5 µl) was spotted on MALDI-TOF disposable target plates (4800, Applied Biosystems, Foster City, CA, USA), then covered with 0.5 µl matrix (100 mM α-cyano-4-hydroxycinnamic acid in 50% acetonitrile and 0.1% trifluoroacetic acid). Peptide mass determinations were carried out using a MALDI-TOF/TOF mass spectrometer (Applied Biosystems 4800 Proteomics Analyzer, Applied Biosystems) in the positive MS reflector mode with the m/z range 900 to 4000. The mass spectrometer is equipped with an Nd∶YAG laser (repetition rate: 200 Hz, λ = 355 nm, pulse width: 5 ns). The parameters of peak detection are: minimum S/N, 10; local noise window width mass/charge (m/z), 250; minimum full-width half-maximum (bins) of 2.9. The instrument was calibrated using the mass standards kit for calibration of AB SCIEX TOF/TOF instruments [M+H]^+^: 904.468 Da, 1296.685 Da, 1570.677 Da, 2093.086 Da, 2465.198 Da.A maximum of the ten strongest monoisotopic precursors per sample were chosen for tandem mass spectrometry (MS/MS) analysis. For protein identification, a combination of peptide mass fingerprints and peptide fragmentation patterns were used. All MS and MS/MS spectra of trypsin digested peptides were automatically processed using Data Explorer version 4.9 software (Applied Biosystems, MDS. Sciex) with the default parameters against protein sequences from the NCBI nonredundant database using the Taxonomy Viridiplantae (Green Plants; 9915903 sequences, 3383666984 residues) and our in-house *A.annua* EST database (2060880 sequences, 49389486 residues) using the following parameters: peptide mass tolerance, 50 ppm; fragment mass tolerance, ±0.1 Da missed cleavages, 1; allowed variable modifications, oxidation (Met). The algorithm provided by the Mascot Engine was adopted [15]. Ions score is −10*Log(P), where P is the probability that the observed match is a random event. Individual ions with scores >41 indicate identity or extensive homology (p<0.05). Protein scores are derived from ions scores as a non-probabilistic basis for ranking protein hits [15]. Proteins that returned a Mascot score exceeded the minimum significant score (NCBI database>71; EST database>76) were considered as a positive identification.

## Results and Discussion

### Isolation of glandular trichomes

**Figure 1:**
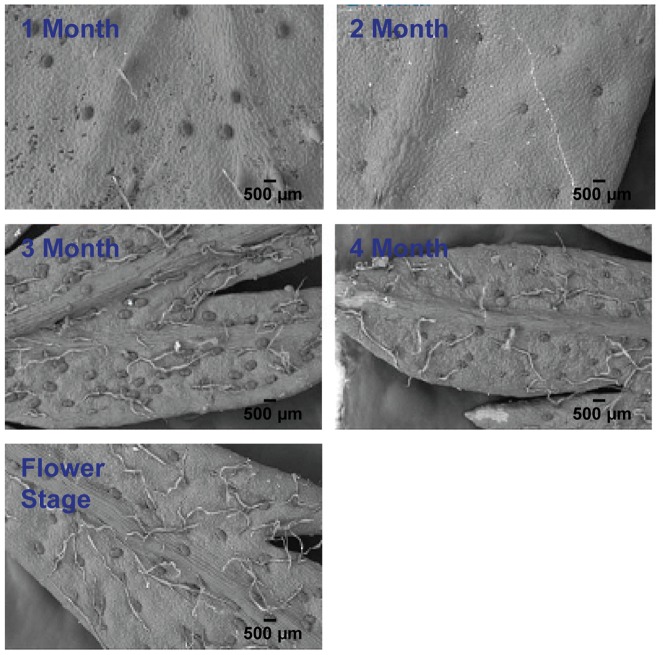
Trichome distribution on the leaf surface of *A. annua* during different developmental stages.

**Figure 2:**
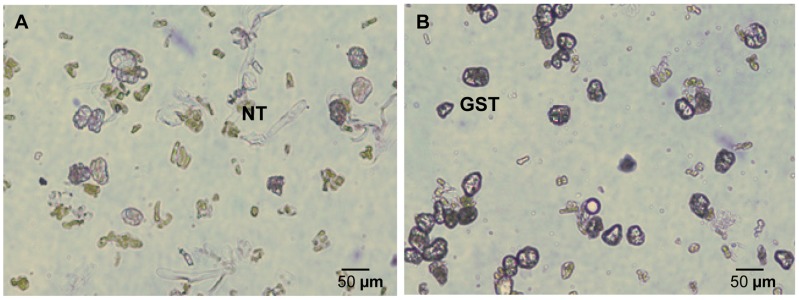
Isolated glandular trichomes recovered from 30 µm mesh (A) and sucrose gradient purification (B). NT, non-glandular trichome; GST, glandular secreting trichome.

Trichome isolation is a critical step in constructing the *A. annua* GST protein database. Two important factors may affect the successful isolation of trichomes: harvest time and isolation method. According to our previous study, which is consistent with previous reports [16], [17], the density of glandular trichomes on the surface of leaf increases dramatically from the 3rd month and remains stable till the inflorescences stage (Fig. 1). Thus, 4-month old plants were chosen for GST isolation. Bead beater abrasion methods have been used to isolate the trichomes from mint and tobacco leaves [13], [18]. However, the leaves of *A. annua* are generally small and deeply dissected, and the GSTs are slightly embedded into the leaf surface. This has made it difficult for the glass beads to reach the base of GSTs. In a test experiment, we found that the quality of GSTs isolated using a bead beater was low and they are usually contaminated with a higher percentage of mesophyll or cell debris (Fig. 2A). 2DE patterns of proteins from leaves and trichomes had no discernible difference (Fig. S1) and over 50% of identified proteins were Rubisco (data not shown). Therefore, an additional sucrose gradient was attempted for further purification after the mechanical abrasion and filtration. A range of sucrose concentrations and different centrifuge speed were tested. Ultimately a 40% sucrose solution and centrifugation of at least 750 rcf yielded preparations with >90% GSTs and no T-shaped trichomes (Fig. 2B). The quality of the preparation was appropriate for protein extraction.

### Identification of proteins in glandular trichcomes

**Figure 3:**
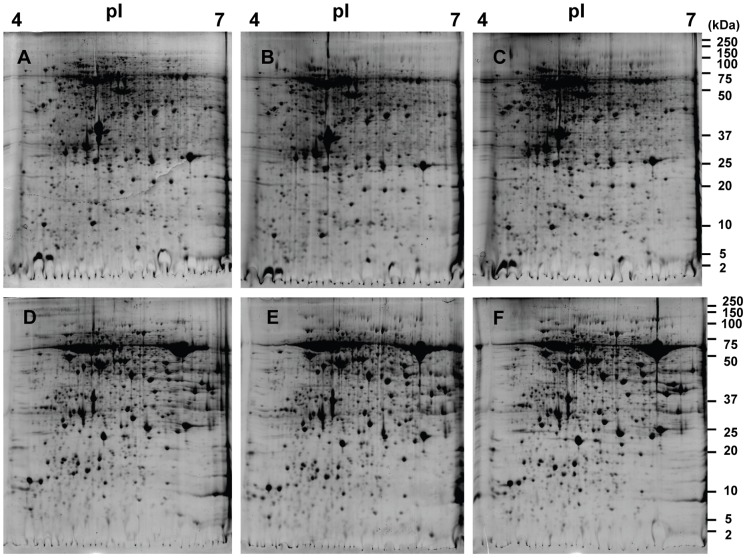
2DE patterns of proteins from trichomes (A, B, C) and leaves (D, E, F). 900 µg of proteins were loaded in each gel and stained with “Blue silver” staining solution. Proteins of isolated glandular trichomes of leaves (A, B, C) and corresponding leaves (D, E, F) are separated on pH gradients 4–7. Three biological repeats were obtained in the study.

In order to identify trichome-specific proteins, the 2-D gel patterns of the leaves and glandular trichomes proteins were compared. As shown in Fig. 3, distinct gel patterns were observed. On “silver blue” stained gels, over 1000 distinct protein spots from leaves and glandular trichomes were separated at pI 4–7. Three biological repeats were used to extract proteins, and highly reproducible results were obtained (95% of protein spots were located at the same position on the gels for leaves and glandular trichomes (Fig. 3). Protein spots (including those up-regulated in trichomes) from glandular trichomes were excised from the gel for MS analysis.

**Figure 4:**
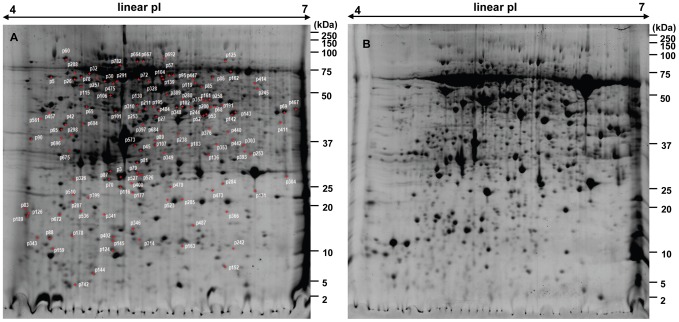
2DE patterns of trichome (A) and leaves (B) proteins. 82 non-redundant proteins in Table S1 were shown on the gel of glandular trichomes. About 70% of them were highly expressed in the glandular trichomes.

Of over 1000 spots on the 2DE gel (Fig. 4A), 738 spots were excised and processed for analysis by MS. Resultant MSMS (assuming not MS only) data were used to interrogate both the NCBI database using the Taxonomy Viridiplantae (Green Plants) and our in-house EST database for protein identifications. Out of the 738 spots, 206 spots (28%) were identified in the NCBI database, whereas 162 (22%) spots were found in our EST database. Taken together, only fewer than 30% of the protein spots could map to known protein or EST sequences, which represent 93 non-redundant proteins. A large number of spots with different pI/Mr values were attributed to the same protein, due to several possible factors: protein post-translational modification, isozyme variation, protein degradation, alternative splicing and allelic variation of the same protein [19].

**Figure 5:**
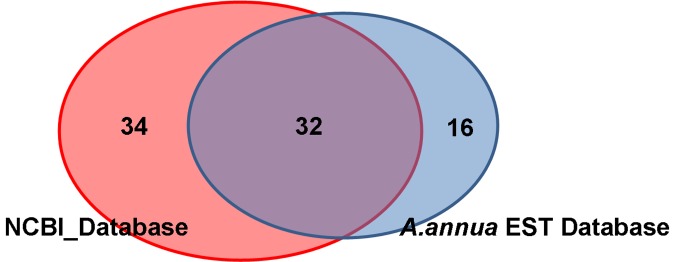
Venn diagram of identified proteins in GSTs using NCBI or Artemisia EST databases.

**Figure 6:**
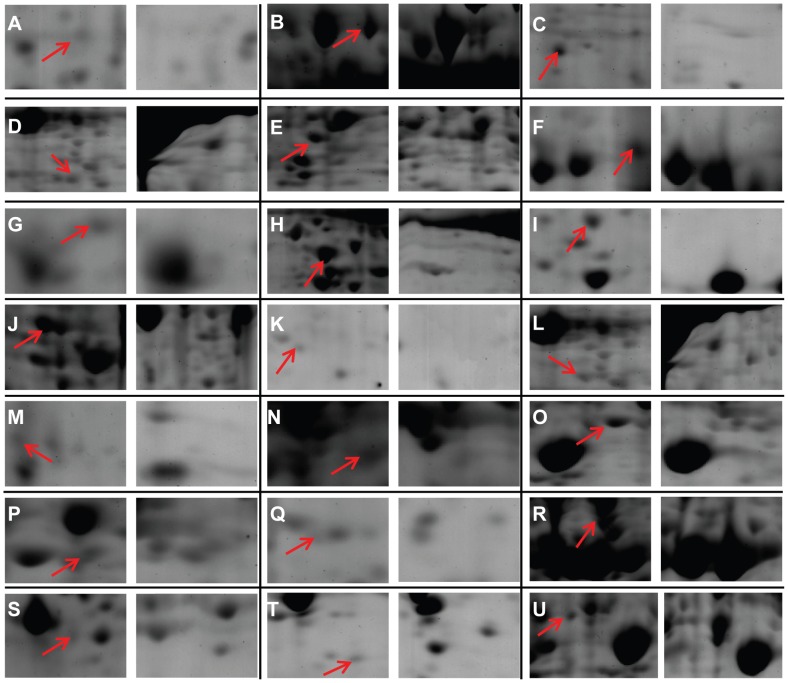
Imagines of up-regulated proteins in glandular trichomes compared with corresponding leaves. The images on the left side of each panel are: A. p510: NAD(P)H-quinone oxidoreductase subunit M (gi|209573110); B. p667: V-type (H+)-ATPase V1, A subunit (gi|224109966); C. p90: cytochrome C oxidase polypeptide vib (gi|168061725); D. p245: artemisinic aldehyde delta-11(13) reductase (gi|197310860); E. p484: plastidic aldolase NPALDP1 (gi|108864048); F. p467: glyceraldehyde-3 phosphate dehydrogenase (gi|115371630); G. p353: phosphoglycerate mutase 1 (gi|114326546); H. p26: phosphopyruvate hydratase (gi|224080171); I. p88: glycine cleavage system H protein (gi|1346119); J. p106: predicted protein (PRK, gi|168006632); K. p242: nucleoside diphosphate kinase B (gi|1346675); L. p414: 4-aminobutyrate aminotransferase (gi|159477247); M. p83: eukaryotic translation initiation factor eIF5A (gi|85376261); N. p328: putative zinc dependent protease (gi|84468324); O. p200: chloroplast FtsH protease (gi|1483215); P. p191: cell division protein FtsH-like protein (072367_3859_2372_2p); Q. p399: cell division protein FtsH-like protein (072367_3859_2372_2p); R: p139: mitochondrial-processing peptidase subunit alpha (gi|266567); S. p457: cysteine protease (gi|239937266); T. p473: peroxiredoxin (Contig38348_2p_5); U. p244: ABC_NikE_OppD_transporters (gi|218196275). The images on the right side of each panel are 2DE gels for corresponding leaves.

Among these 93 non-redundant proteins, the most abundant ones are those involved in photosynthesis (e.g. oxygen-evolving enhancer protein, ribulose-1,5- bisphosphate carboxylase, chloroplast light-harvesting chlorophyll a/b-binding protein). This result is consistent with previous reports that the glandular trichomes of *A. annua* contain two pairs of green secretary cells in which photosynthesis occurs [20], [21]. These proteins were omitted in the following analysis. Of the other 82 proteins, 66 and 48 were positively matched to NCBI databases and our in-house EST database respectively (Fig. 4A, Fig. 5). Among the 16 proteins identified exclusively from the Artemisia EST database, a total of 10 proteins have no homolog with proteins from any other species (even when employing loose BLAST parameters), indicating they might be species-specific and/or tissue-specific. Changes in spot intensity between glandular trichomes and the whole leaves were also quantified. Over 70% of the 82 non-redundant proteins were highly expressed in glandular trichomes. Among them, important artemisinin pathway enzymes, e.g, artemisinic aldehyde delta-11(13) reductase (Dbr2), and 2-C-methyl-D-erythritol 2, 4-cyclodiphosphate synthase (MECDP-synthase) were identified. Both of them are highly enriched in the glandular trichomes. The full list of proteins highly expressed in GSTs and the images of some of up-regulated proteins in GSTs can be found in **Figure 4**, **Figure 6** and **Table S1**, respectively.

### Function annotation of GST-expressed proteins

**Figure 7:**
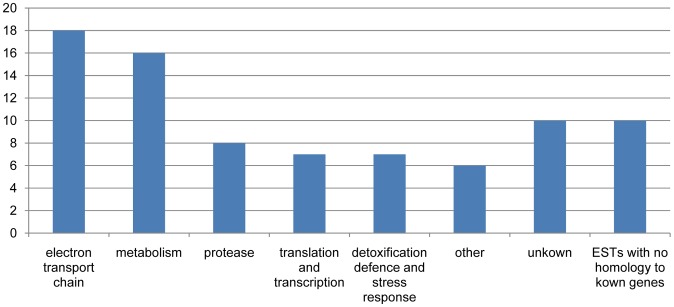
Categorization and distribution of proteins in glandular trichomes.

The 82 identified proteins are classified into the following categories based on the UniProt database (http://www.uniprot.org): electron transport chain, proteolysis, translation and transcription, metabolism, detoxification or defense and stress response, others and unknown function proteins (Table S1; Fig. 7). The annotation was assigned mainly according to the predominant function even if the proteins may play various roles in different subcellular compartments or at different developmental stages. According to our previous transcriptome study on the GSTs extracted from flower buds [11], many genes with high abundant transcript expression also belong to these functional categories.

### 1) Proteins involved in electron transport chain

This class mainly comprises several enzymes in adenosine triphosphatase (ATPase) family, including subunits of the ATP-synthase complex (alpha, beta, gamma, epsilon subunits etc) and V-type (H^+^)-ATPase V1 (p667). The ATPase family is important enzymes involved in energy production and in maintaining ion concentration balance in plant cells. Among them, vacuolar H^+^-ATPase (V-ATPase) is regarded as the basis for salt tolerance by generating proton gradients to control ion extrusion and pH regulation [22], [23]. The enhancement of these proteins in several plants exposed to cold stress implies that more energy is needed to reinforce plant self-defense system to cold stress [24], [25]. Furthermore, other enzymes including ferredoxin-NADP reductase (p52), NAD(P)H-quinone oxidoreductase (p510), cytochrome c oxidase subunit Vib (p90), ATP binding/succinate dehydrogenase (p125) were identified in GSTs. These enzymes have been well characterized in plant mitochondria or chloroplasts and some of them are thought to function in protecting plant from oxidative stress by catalyzing electron transport in the respiratory chain [26]–[28]. In addition, ferredoxin-NADP reductase also participates in various metabolic pathways, including terpenoid biosynthesis, steroid metabolism [29]. The increased abundance of some of these proteins in glandular trichomes of *A. annua* is consistent with previous study that high energy is demanded for powering other cellular reactions in glandular trichomes, such as isoprenoid or other carbon fixation, fatty acid metabolism [30].

### 2) Proteins involved in metabolism

Another large class representing proteins involved in metabolic process, e.g. artemisinin biosynthesis, glycolysis, and other catalytic function (e.g. carbon fixation, glycine degradation).

**Figure 8:**
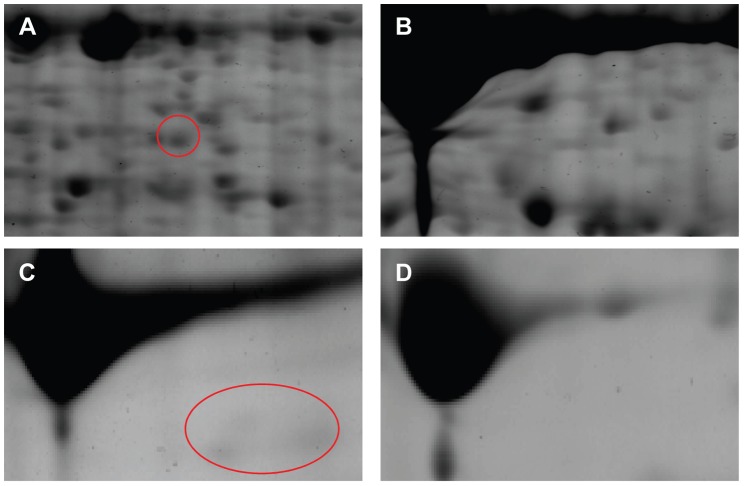
Enlarged 2DE gels for isolated trichomes (A, C) and corresponding leaves (B, D). The labeled protein spots showed a higher protein accumulation in the isolated trichomes compared with the corresponding leaf tissue. A, B: DBR2; C, D: MECDP-synthase.

**Figure 9:**
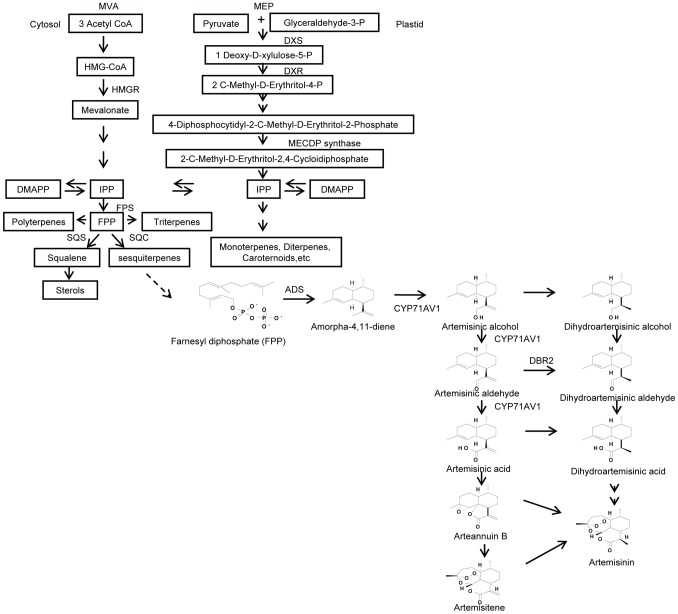
The simplied pathway of terpenoid biosynthsis in plants. Modified from reported data [48] MVA, 3R-Mevalonic acid; MEP, 2-C-Methy-D-erythritol 4-phosphate; DMAPP, dimethylallyl diphosphate; IPP, isopentenyl diphosphate; FPS, farnesyl diphosphate synthase; HMGR, 3-hydroxy-3-methylglutaryl coenzyme A reductase; DXS, 1-deoxy-D-xylulose-5-phosphate synthase; DXR, 1-deoxy-D-xylulose-5-phosphate reductoisomerase; SQC, sesquiterpene cyclase; SQS, squalene synthase.

Artemisinic aldehyde delta-11(13) reductase (Dbr2, p245, Fig. 8A) was first characterized in vitro by Zhang and his coworkers [6]. It belongs to an enzyme family that acts on α, β-unsaturated carbonyls and presents the subfamily associated with terpene double bond reduction, the FMN-linked oxidoreductases. It is a trichome specific enzyme involved in the sesquiterpenoid Δ11(13) double bond reduction to the conversion of artemisinic aldehyde to dihydroartemisinic aldehyde (Fig. 9). Another protein has a blast hit to MECDP-synthase (Fig. 8C), another important enzyme involved in terpenoid biosynthesis. MECDP-synthase belongs to the lyases family, specifically phosphorus-oxygen lyases which catalyzes the conversion of 2-phospho-4-(cytidine 5′-diphospho)-2-C-methyl-D-erythritol to 2-C-methyl-D- erythritol 2, 4-cycloidi phosphate (MECDP) accompanying cytidine monophosphate (CMP) generation. Terpenoids can be derived from the cytosolic mevalonate (MVA) pathway or from the plastidial 2-Cmethyl-D-erythritol-4-phosphate (MEP) pathway [31], [32]. Both pathways lead to the formation of the C5 units isopentenyl diphosphate (IPP) and its allylic isomer dimethylallyl diphosphate (DMAPP), the basic terpenoid biosynthesis building blocks. It has long been assumed that artemisinic acid is a direct precursor of artemisinin. However, recent feeding experiments with artemisinic acid [33] and dihydroartemisinic acid [34] have shown that the latter substance is the precursor of artemisinin. Evidence also suggests that the plastidial MEP pathway may also be involved in precursor supply for artemisinin biosynthesis which occurs in the cytoplasm [35]. MECDP synthase participates in the terpenoid biosynthesis in MEP pathway, leading to the formation of IPP, the precursor for artemisinin and a wide range of other terpenoids (Fig. 9). The gene encoding this enzyme has been cloned from *Ginkgo biloba* [36] and the x-ray crystal structure of MECDP synthase has been characterized [37]. In our study, both of these two enzymes can only be detected in GSTs (Fig. 8). However, high levels of MEP pathway enzymes cannot be directly associated with artemisinin biosynthesis without further investigation.

Among the identified proteins, 4 encode enzymes implicated in glycolysis. They are fructose-1, 6-bisphosphate adolase (p484), glyceraldehyde-3 phosphate dehydrogenase (p467), phosphoglycerate mutase (p353), phosphopyruvate hydratase (p26). Glycolytic enzymes are considered as important suppliers of essential metabolic flexibility that facilitates plant development and acclimation to environmental stress. They convert glucose into pyruvate and provide essential energy to cells. At the same time, a large number of carbons in the plant glycolytic pathways are utilized in the biosynthesis of numerous compounds, such as isoprenoids, amino acids, nucleic acids, and fatty acids especially important to the autotrophic tissues [38]. In *A. annua*, the intermediate product glyceraldehyde 3-phosphate and final product pyruvate participate in the formation of IPP and DMAPP, the precursors for the synthesis of artemisinin and other monoterpenes or isoprenoids [39]. Changes of glycolytic enzymes have been reported in plant and non-plant species against environment stresses including nutrient limitation, salt, drought, and radioactivity [40]–[41] [42]. However, the mechanism is not yet fully elucidated.

Other enzymes with different catalytic functions include phosphoribulokinase (p106), nucleoside diphosphate kinase (p242), 4-aminobutyrate aminotransferase (p414) etc. These enzymes participate in carbon fixation (phosphoribulokinase), glycine degradation (glycine cleavage system H protein), or in maintaining equilibrium between the concentrations of different nucleoside triphosphates (nucleoside diphosphate kinase).

### 3) Proteins involved in transcription and translation

Proteins involved in gene transcription (messenger ribonucleic acid (mRNA) synthesis) and mRNA translation (protein synthesis) were increased in GSTs compared to leaves. Changes in the levels of these enzymes can be attributed to active role of GSTs in the metabolites biosynthesis and protection of plant against biotic or abiotic stress, which requires new proteins and RNA synthesis. Among them, eukaryotic translation initiation factor eIF5A (p83) is a highly conserved protein functions in the transport of a subset of mRNAs out of the nucleus to the ribosome. It is mainly active in translation elongation, mRNA turnover and decay, regulation of cell division, cell growth and cell death. However, its function in high plant is unclear. Recent reports found that eIF5A was up-regulated in Arabidopsis root under Fe deficiency conditions. They speculated that eIF5A might play important role in regulating translation under stress conditions and in adapting plants to prevailing environmental conditions [43].

### 4) Proteins involved in proteolysis

Several enzymes involved in proteolysis are also identified in this study. These include zinc dependent protease (p328), chloroplast FtsH protease (p200), cell division protein FtsH-like protein (p191), cell division protein ftsH (p399), predicted Zn-dependent peptidases (p139), 20S proteasome alpha subunit A (p442), cysteine protease (p457). FtsH, a member of AAA^+^ ATPases, is membrane bound ATP dependent metalloprotease which functions in assembly, operation and disassembly of protein complexesjavascript:void(0);. Changes of FstH following cold acclimation in some plants suggests that the FtsH ATPases may function in chloroplast or plasma–membrane, and post translational modification of cold responsive proteins may be a basis for cold tolerance in plants response to cold stress [44], [45]. In addition, FtsH has been reported to participate in cell division in *Bacillus subtilis* [46].The differential expression of several FstH proteins between glandular trichomes and leaves reinforce the fact that GSTs play important roles in plant defense against severe environmental conditions.

### 5) Detoxification, stress defense related proteins

A variety of proteins associated with detoxification, stress defense were also identified. This group of proteins includes dehydration stress-induced protein (p126), rhodanese homology domain (p285), peroxiredoxin (p473), glycine-rich RNA-binding protein (p346), ferritin (p349), ABC_NikE_OppD_ transporters (p244). One of the most important proteins in this group is ABC_NikE_OppD_ transporters. ATP-binding cassette transporters (ABC transporters) are one of the largest transporter families found in prokaryotes and eukaryotes. They couple ATP hydrolysis to transport a wide variety of substrates such as ions, amino acids, peptides, sugars, lipids, and sterols, across cell membranes. Plant ABC transporters were first reported to be involved in detoxification. However, they have recently been implicated to function in ion regulation and plant growth process etc [47]. The ABC_NikE_OppD_transporters identified in our study is associated with the transport of dipeptides, oligopeptides (OppD), and nickel, which may be essential in protecting plants against toxin accumulation. Several proteins with unique or unknown biological functions are also listed in Figure 6. Further investigation is needed in order to understand their roles in GSTs.

In conclusion, enriched but inter-species un-conserved genes identified in this study, together with our previous transcriptome study suggest that more trichome-specific genes involved in Artemisia metabolism and trichome development are present than previously thought. These candidate proteins provide potential targets for further elucidation of trichome function and artemisinin metabolism in the important medicinal plant *A.annua*.

## Supporting Information

Figure S1 **2-DE patterns of leaf (A) and trichomes (B) of Artemisia proteins.** Trichomes were isolated were isolated from leaves of *A. annua* plants using the bead beater method as described previously [49]. No sucrose enrichment was carried out. After abrasion by a bead beater machine (BioSpec Products, Inc., Bartlesville, OK, USA), the crude cellular extract was separated by sequentially filtering through a 105, 40 and 30 µm nylon mesh (Small Parts Inc., Miami Lake, FL, USA). Then proteins were extracted as described in materials and methods. The protein lysate was further purified by 2-D Clean-Up kit (GE Healthcare, USA). Then 700 mg of proteins was loaded in each gel and stained with “Blue silver” staining solution. Proteins were separated on pH gradients 4–7 and further subjected to 2DE and MALDI-TOF/TOF MS analysis as described in materials and methods.
(TIF)

Table S1 **identified proteins in glandular trichomes.**
(DOC)
