# Supplementary figures and images for: Investigation of Glandular Trichome Proteins in Artemisia annua L. Using Comparative Proteomics
Source: PLoS One. 2012 Aug 8;7(8):e41822. doi: 10.1371/journal.pone.0041822 (PMC3414485; doi:10.1371/journal.pone.0041822)

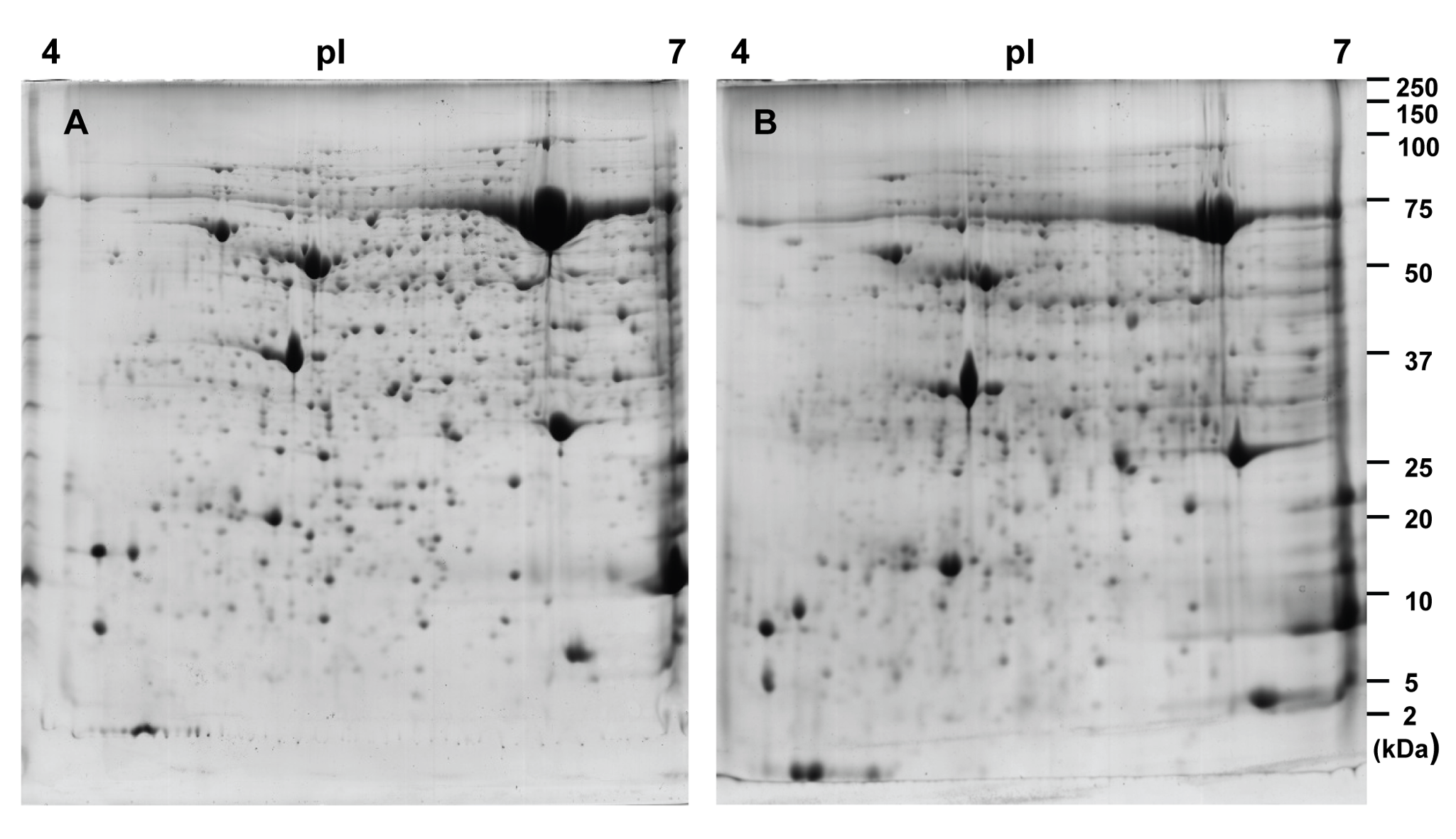

Supplement: Figure S1 — 2-DE patterns of leaf (A) and trichomes (B) of Artemisia proteins. Trichomes were isolated were isolated from leaves of A. annua plants using the bead beater method as described previously [49]. No sucrose enrichment was carried out. After abrasion by a bead beater machine (BioSpec Products, Inc., Bartlesville, OK, USA), the crude cellular extract was separated by sequentially filtering through a 105, 40 and 30 µm nylon mesh (Small Parts Inc., Miami Lake, FL, USA). Then proteins were extracted as described in materials and methods. The protein lysate was further purified by 2-D Clean-Up kit (GE Healthcare, USA). Then 700 mg of proteins was loaded in each gel and stained with “Blue silver” staining solution. Proteins were separated on pH gradients 4–7 and further subjected to 2DE and MALDI-TOF/TOF MS analysis as described in materials and methods. (TIF) [file pone.0041822.s001.tif]
