# Supplementary material for: Investigation of Glandular Trichome Proteins in Artemisia annua L. Using Comparative Proteomics
Source: PLoS One. 2012 Aug 8;7(8):e41822. doi: 10.1371/journal.pone.0041822 (PMC3414485; doi:10.1371/journal.pone.0041822)
Supplement: Table S1 — identified proteins in glandular trichomes. (DOC) [file pone.0041822.s002.doc]

| **Table S1 identified proteins in glandular trichomes** | | | | | | | | | | | | | | | | | | | | | | | | | | | | | | | | | | | | | | | | | | | | | | | | | | | | | | | | | | | | | | | | | | | | | | | | | | | | | | | | | | | | | | | | | | | | | | |  | | | | | | |
| --- | --- | --- | --- | --- | --- | --- | --- | --- | --- | --- | --- | --- | --- | --- | --- | --- | --- | --- | --- | --- | --- | --- | --- | --- | --- | --- | --- | --- | --- | --- | --- | --- | --- | --- | --- | --- | --- | --- | --- | --- | --- | --- | --- | --- | --- | --- | --- | --- | --- | --- | --- | --- | --- | --- | --- | --- | --- | --- | --- | --- | --- | --- | --- | --- | --- | --- | --- | --- | --- | --- | --- | --- | --- | --- | --- | --- | --- | --- | --- | --- | --- | --- | --- | --- | --- | --- | --- | --- | --- | --- | --- | --- | --- | --- | --- | --- | --- | --- | --- | --- | --- |
| **Spot ID** | | | | | **Changes*^a^*** | | | | | | | | | | | | | | | | | | **pI/Mr** | | | | | | | **Best protein score*^b^*** | | | | | | | | | | | | | | | | | | | | | | | | **Homolog_GI** | | | | | | | | | | | | | **Protein Name** | | | | | | **EST best protein score** | | | | | | | | | | | | | | | | | | | | | **Best matching EST ID** |  | | | | | | |
| **Electron transport chain** | | | | | | | | | | | | | | | | | | | | | | | | | | | | | | | | | | | | | | | | | | | | | | | | | | | | | | | | | | | | | | | | | | | | | | | | | | | | | | | | | | | | | | | | | | | | | | |  | | | | | | |
| p684|p527|p95|p136|p79 | | | | | | | | | | | | up | | | | | | | | | | | 5.20/55482 | | | | | | | | | | | | 472 | | | | | | | | | | | | | gi|94502485,gi|114522,gi|115502358 | | | | | | | | | | | | | | | | | | ATP synthase CF1 alpha subunit [Helianthus annuus] | | | | | | | | | | | | 108 | | | | | | | | | | 092121_2539_3868_3r | | | | | | | |  | | | | | |
| p314|p101|p89|p163 | | | | | | | | | | | | up | | | | | | | | | | | 5.42 /13772 | | | | | | | | | | | | | | 443 | | | | | | | | | | | gi|81176256,gi|22795943 | | | | | | | | | | | | | | | | | | ATP synthase CF1 epsilon subunit [Lactuca sativa] | | | | | | | | | | | | 115 | | | | | | | | | | | 132965_1015_3960_2p_3,Contig8503_3p_21 | | | | | | | |  | | | | |
| p402|p145 | | | | | | | | | | | | up | | | | | | | | | | | 5.42 /13772 | | | | | | | | | | | | 358 | | | | | | | | | | | | | gi|6635347 | | | | | | | | | | | | | | | | | | ATPase epsilon chain [Lactuca sativa] | | | | | | | | | | | | 94 | | | | | | | | | | | | | Contig16137_3p_17 | | | |  | | | | | | |
| p125 | | | | | | | | | | | |  | | | | | | | | 5.86 /69612 | | | | | | | | | | | | | | | 239 | | | | | | | | | | | | | gi|15240075 | | | | | | | | | | | | | | | | | | SDH1-1; ATP binding / succinate dehydrogenase [Arabidopsis thaliana] | | | | | | | | | | | | | | | | | |  | | |  | | | | | | | | | |  | | | | |
| p195|p280|p65|p696|p72***^△^*** | | | | | | | | | | | | up | | | | | | | | | | | 5.11/52197 | | | | | | | | | | | | | 384 | | | | | | | | | | | | gi|5758861,gi|7708518,gi|153012228,gi|241912832 | | | | | | | | | | | | | | | | | | ATP synthase CF1 beta subunit [Medicago truncatula] | | | | | | | | | | | | 391 | | | | | | Contig8503_1p_4,Contig2065_3p_1,Contig14249_3r | | | | | | | | | | | | | |  | | | |
| p3|p30 | | | | | | | up | | | | | | | | | 5.04/33964 | | | | | | | | | | | | | | | | | | | | | | | | | 134 | | | | | gi|224159128 | | | | | | | | | | | | | | | | | | | | predicted protein [Populus trichocarpa] | | | | | | |  | | | | | | | | | | |  | | | | | | | | | | | | | |  | | | |
| p57 | | | | | | | up | | | | | | | | | 7.87/213765 | | | | | | | | | | | | | | | | | | | | | | | | | 350 | | | | | gi|218194450 | | | | | | | | | | | | | | | | | | | | hypothetical protein OsI_15081[Oryza sativa Indica Group] | | | | | | | | | | | | | | | |  | | |  | | | | | | | | | | | | | | | |  |
| p440|p238|p162 | | | | | | | up | | | | | | | | | 6.02/55452 | | | | | | | | | | | | | | | | | | | | | | | | | 440 | | | | | gi|114404,gi|20146574 | | | | | | | | | | | | | | | | | | | | ATP synthase subunit alpha, mitochondrial | | | | | | | | | | | | 181 | | | 141790_3502_1279_3r_1,Contig35597_2p_6 | | | | | | | | | | | | | | | | |  | | | |
| p104 | | | | | | | | | |  | | | | | | 5.63/20241 | | | | | | | | | | | | | | | | | | | | | | | | | 157 | | | | | gi|210076131 | | | | | | | | | | | | | | | | | | | | mitochondrial ATP synthase beta subunit [Dimocarpus longan] | | | | | | | | | | | | 132 | | | 048139_1764_1521_1r | | | | | | | | | | | | | | | | |  | | | |
| p161|p258 | | | | | up | | | | | | | | | | | | | | | 5.95/40049 | | | | | | | | | | | | | | | | | 175 | | | | | | | | | | | gi|83777062 | | | | | | | | | | | | | | | | | | ATP synthase gamma subunit [Phaseolus vulgaris] | | | | | | | | | | 233 | | | | | | | | Contig13579_3p_4 | | | | | | | | | | | | | | |  | | |
| p782|p102 | | | | | | | up | | | | | | | | | 5.14/55538 | | | | | | | | | | | | | | | | | | | | | | | | | 169 | | | | | gi|225465149 | | | | | | | | | | | | | | | | | | | | PREDICTED: hypothetical protein [Vitis vinifera] | | | | | | |  | | | | | | | | | | |  | | | | | | | | | | | | | |  | | | |
| p70 | | | |  | | | | | | | | | | | | | | | | | | | 8.98/26768^c^ | | | | | | | | | 104 | | | | | | | | | | | | | | | | | | | | gi|416681 | | | | | | | | | | | | | | ATP synthase delta chain, chloroplastic | | | | | | | | | | | | 110 | | | Contig4565_2p_3 | | | | | | | | | | | | | | | | | | | | |
| p667 | | | | | | | up | | | | | | | | | | 5.29/68792 | | | | | | | | | | | | | | | | | | | | | | | | 331 | | | | | gi|224109966 | | | | | | | | | | | | | | | | | | | | V-type (H+)-ATPase V1, A subunit | | | | | | | | | | | | 144 | | | Contig5858_1r | | | | | | | | | | | | | | | | | | |  | |
| p52|p53|p142|p143 | | | | | | | | | | |  | | | | | | 8.37/40419 | | | | | | | | | | | | | | | | | | | | | | | | 227 | | | | | gi|61969078,gi|222423270 | | | | | | | | | | | | | | | | | | | | putative ferredoxin-NADP reductase [Lycopersicon peruvianum] | | | | | | | | | | | | 136 | | | 026370_1594_0779_2p_1,214374_0319_3906_3r_1,Contig23006_1p | | | | | | | | | | | | | | | | | | |  | |
| p510 | | | | | | | | up | | | | | | | | | | 5.10/23523 | | | | | | | | | | | | | | | | | | | | | | | | 138 | | | | | gi|209573110 | | | | | | | | | | | | | | | | | | | NAD(P)H-quinone oxidoreductase subunit M | | | | | | |  | | | | | | | | | | |  | | | | | | | | | | | | | |  | | | |
| p178 | | | | | | | | up | | | | | | | | | | 9.19/64123 | | | | | | | | | | | | | | | | | | | | | | | | 111 | | | | | gi|255548890 | | | | | | | | | | | | | | | | | | | hypothetical protein RCOM_0923760 [Ricinus communis] | | | | | | |  | | | | | | | | | | |  | | | | | | | | | | | | | |  | | | |
| p90* | | | | | | | | up | | | | | | | | | | NA^d^ | | | | | | | | | | | | | | | | | | | | 86 | | | | | | | | | | | gi|168061725 | | | | | | | | | | | | | | | | | cytochrome C oxidase polypeptide vib | | | | | | | | | | | | 138 | | | | | | Contig22469_3p_3 | | | | | | | | | | | | | |  | | | |
| p400|p177|p284|p341|p116 | | | | | | | | | | | | | up | | 5.31/19707 | | | | | | | | | | | | | | | | | | | | | | | | | | | | | 176 | | | gi|192910736 | | | | | | | | | | | | | | | | | | | mitochondrial F0 ATP synthase D chain[Elaeis guineensis] | | | | | | | | | | | | 338 | | | | | | Contig3641_2p_4 | | | | | | | | | | | | | |  | | | |
| **Translation and Transcription** | | | | | | | | | | | | | | | | | | | | | | |  | | | | | | | | | | | |  | | | | | | | | | | | | | | |  | | | | | | | | | | |  | | | | |  | | | | | | |  | | | | | | | | | | |  | | | | | | | | | | | | | |  | | | |
| p692 | | | | | | | | | up | | | | | | | | | 4.63/37643 | | | | | | | | | | | | | | | | | | | | | | | | 323 | | | | | gi|224172764 | | | | | | | | | | | | | | | | | | | predicted protein [Populus trichocarpa] | | | | | | |  | | | | | | | | | | |  | | | | | | | | | | | | | |  | | | |
| p672 | | | | | | up | | | | | | | | | | | | | | | 6.34/20309 | | | | | | | | | | | | | | | | | 77 | | | | | | | | | | | | gi|133088 | | | | | | | | | | | | | | | | 50S ribosomal protein L12 | | | | | | | | | | | | 72 | | | | | | Contig33808_1p_2 | | | | | | | | | | | | | |  | | | |
| **(Continued)** | | | | | | | | | | | | | | | | | | | | | | | | | | | | | | | | | | | | | | | | | | | | | | | | | | | | | | | | | | | | | | | | | | | | | | | | | | | | | |  | | | | | |  | | | | | | | | | | | | | |  | | | |
| **Spot ID** | | | | | **Changes*^a^*** | | | | | | | | | | | | | | | | | | | **pI/Mr** | | | | | | | | | **Best protein score*^b^*** | | | | | | | | | | | | | | | | | | | | | | | **Homolog_GI** | | | | | | | **Protein Name** | | | | | | | | | **EST best protein score** | | | | | | | | | | | | | | | | | | | | | **Best matching EST ID** | | | | |  | | | |
| p183 | | | | | | up | | | | | | | | | | | | | 7.05/29063 | | | | | | | | | | | | | | 156 | | | | | | | | | | | | | | | | | | | | | gi|82621186 | | | | | | | | | | | | transcription factor APFI-like [Solanum tuberosum] | | | | | | | | 94 | | | | | | | | | | Contig31551_1p_1 | | | | | | | | | | | | | |  | | | |
| p5 | | | | | | | | |  | | | | | | | | | | 5.15/2643 | | | | | | | | | | | | | | | 103 | | | | | | | | | | | | | | | | | | | | gi|399305 | | | | | | | | | | | | Calreticulin | | | | | | | | 87 | | | | | | | | | | Contig33337_2r_2 | | | | | | | | | | | | | |  | | | |
| p69 | | | | | | up | | | | | | | | | | | | | 5.15/172051 | | | | | | | | | | | | 81 | | | | | | | | | | | | | | | | | | | | | | | | gi|196128985 | | | | | | | | | | RNA polymerase beta' chain [Chlorella vulgaris] | | | | | | | |  | | | | | | | | | | |  | | | | | | | | | | | | | |  | | | |
| p291 | | | | | | up | | | | | | | | | | | | | 5.41/12112 | | | | | | | | | | | | 110 | | | | | | | | | | | | | | | | | | | | | | | | gi|160895751 | | | | | | | | | | nitrogen regulatory protein P-II GLUTAMINE | | | | | |  | | | | | | | | | | | | | |  | | | | | | | | | | | | | | | | |
| p83 | | | | | | up | | | | | | | | | | | | | 5.30/17121 | | | | | | | | | | | | 88 | | | | | | | | | | | | | | | | | | | | | | | | gi|85376261 | | | | | | | | | | eukaryotic translation initiation factor eIF5A | | | | | | | | | | 98 | | | | | | | | 127634_0717_1720_3r | | | | | | | | | | | | | | | | | | |
| **Metabolism** | | | | | | | | | | | | | | | | | | | | | | | | |  | | | | | | | | | | | | | |  | | | | | | | | | | | |  | | | | | | | | | | |  | | |  | | | | | | | | | |  | | | | | | | | | | |  | | | | | | | | | | | | | | | |
| p60 | | | | | | up | | | | | | | | | | | | | 5.20/72856 | | | | | | | | | | | | 145 | | | | | | | | | | | | | | | | | | | | | | | | gi|168044813 | | | | | | | | | | predicted protein [Physcomitrella patens subsp. patens] | | | | | | | | | |  | | | | | | | | | | |  | | | | | | | | | | | | | | | |
| p479 | | |  | | | | | | | | | | | | | | | | | | | NA | | | | | | | | |  | | | | | | | | | | | | | | | | | | | | | |  | | | | | | | | | | | | mangrin [Bruguiera sexangulamangrin] | | | | | | | | | | 89 | | | | | | | | Contig6064_1r_2 | | | | | | | | | | | | | | | | | | |
| p88 | | | | | | up | | | | | | | | | | | | | 5.04/134619 | | | | | | | | | | | | 76 | | | | | | | | | | | | | | | | | | | | | | | | gi|1346119 | | | | | | | | | | Glycine cleavage system H protein | | | | | | | | | |  | | | | | | | | | | |  | | | | | | | | | | | | | | | |
| p106 | | | | | | up | | | | | | | | | | | | | 4.78/22695 | | | | | | | | | | | | 97 | | | | | | | | | | | | | | | | | | | | | | | | gi|168006632 | | | | | | | | | | predicted protein (PRK) | | | | | | | | | | 90 | | | | | | | | Contig40843_3p_1 | | | | | | | | | | | | | | | | | | |
| p487 | | | | | | | | |  | | | | | | | | | | 9.5/123954 | | | | | | | | | | | | 286 | | | | | | | | | | | | | | | | | | | | | | | | gi|229473559 | | | | | | | | | | nucleoside diphosphate kinase II | | | | | | | | | | 133 | | | | | | | | 097220_0761_3711_3r_1 | | | | | | | | | | | | | | | | | | |
| p242 | | | | | | up | | | | | | | | | | | | | 6.43/16189 | | | | | | | | | | | | 235 | | | | | | | | | | | | | | | | | | | | | | | | gi|1346675 | | | | | | | | | | Nucleoside diphosphate kinase B | | | | | | | | | | 161 | | | | | | | | 316948_2128_2485_1p_1 | | | | | | | | | | | | | | | | | | |
| p484| p27 |p207|p211|p348 | | | | | | | | | | | | | | up | 6.92 /42547 | | | | | | | | | | | | | | | | | | | 174 | | | | | | | | | | | | | | | | | | | | gi|108864048,gi|4827251 | | | | | | | | | | | plastidic aldolase NPALDP1 [Nicotiana paniculata] | | | | | | | | | | | | 115 | | | Contig6205_2p,344418_1450_0111_3r_1,Contig20966_1p_2 | | | | | | | | | | | | | | | | | | | | | |
| p467 | | | | | | | | | up | | | | | | 8.30/36662 | | | | | | | | | | | | | | | | | | | 80 | | | | | | | | | | | | | | | | | | | | | gi|115371630 | | | | | | | | | | glyceraldehyde-3 phosphate dehydrogenase | | | | | | | | | |  | | | | | | | | | | |  | | | | | | | | | | | | | | | |
| p353 | | | | | | | | | up | | | | | | 6.67/28814 | | | | | | | | | | | | | | | | | | | 80 | | | | | | | | | | | | | | | | | | | | | gi|114326546 | | | | | | | | | | phosphoglycerate mutase 1 | | | | | | | | | | | |  | | | | | | | | |  | | | | | | | | | | | | | | | |
| p310 | | | | | | up | | | | | | | | | | | | | 5.90/48165 | | | | | | | | | | | | 73 | | | | | | | | | | | | | | | | | | | | | | | | | gi|194702114 | | | | | | | | | unknown [Zea mays] | | | | | | | | | |  | | | | | | | | | | |  | | | | | | | | | | | | | | | |
| p26 | | | | | | up | | | | | | | | | | | | | 6.06/24549 | | | | | | | | | | | | 82 | | | | | | | | | | | | | | | | | | | | | | | | | gi|224080171 | | | | | | | | | phosphopyruvate hydratase | | | | | | | | | |  | | | | | | | | | | |  | | | | | | | | | | | | | | | |
| p414 | | | | | | up | | | | | | | | | | | | | 6.59/43596 | | | | | | | | | | | | 107 | | | | | | | | | | | | | | | | | | | | | | | | | gi|159477247 | | | | | | | | | 4-aminobutyrate aminotransferase | | | | | | | | | |  | | | | | | | | | | |  | | | | | | | | | | | | | | | |
| p376 | | | | | |  | | | | | | | | | | | | | 7.60/27963 | | | | | | | | | | | | 72 | | | | | | | | | | | | | | | | | | | | | | | | | gi|62320917 | | | | | | | | | carbonic anhydrase | | | | | | | | | | 138 | | | | | | | | 140570_0931_3601_3r_1 | | | | | | | | | | | | | | | | | | |
| p526 | | | | | |  | | | | | | | | | | | | | 5.30/36934 | | | | | | | | | | | | 70 | | | | | | | | | | | | | | | | | | | | | | | | | gi|226460634 | | | | | | | | | predicted protein [Micromonas pusilla CCMP1545] | | | | | | | | | |  | | | | | | | | | | |  | | | | | | | | | | | | | | | |
| p85 | | | | | | up | | | | | | | | | | | | | 5.10/114085 | | | | | | | | | | | | 72 | | | | | | | | | | | | | | | | | | | | | | | | | gi|116783747 | | | | | | | | | Nitrilase/cyanide hydratase | | | | | | | | | |  | | | | | | | | | | |  | | | | | | | | | | | | | | | |
| p245 | | | | | | up | | | | | | | | | | | | | 6.13 /42420 | | | | | | | | | | | | 232 | | | | | | | | | | | | | | | | | | | | | | | | | gi|197310860 | | | | | | | | | artemisinic aldehyde delta-11(13) reductase [Artemisia annua] | | | | | | | | | | | | | | 95 | | | | Contig11872_1p_2 | | | | | | | | | | | | | | | | | | |
|  | | | | | | | | | | | | | | | | | | | | | | | | |  | | | | | | | | | | | | | |  | | | | | | | | | | | |  | | | | | | | | | | |  | | |  | | | | | | | | | |  | | | | | | | | | | |  | | | | | | | | | | | | | | | |
| **(Continued)** | | | | | | | | | | | | | | | | | | | | | | | | |  | | | | | | | | | | | | | |  | | | | | | | | | | | |  | | | | | | | | | | |  | | |  | | | | | | | | | |  | | | | | | | | | | |  | | | | | | | | | | | | | | | |
| **Spot ID** | | | **Changes*^a^*** | | | | | | | | | | | | | | | | | | | | | | | | **pI/Mr** | | | | | | | **Best protein score*^b^*** | | | | | | | | | | | | | | | | | | | | | | | **Homolog_GI** | | | | | | | | | | | **Protein Name** | **EST best protein score** | | | | | | | | | | | | | | | | | | | | | **Best matching EST ID** | | | | | | | | | | | |
| **Protease** | | | | | | | | | | | | | | | | | | | | | | | | |  | | | | | | | | | | | | | |  | | | | | | | | | | | |  | | | | | | | | | | |  | | |  | | | | | | | | | |  | | | | | | | | | | |  | | | | | | | | | | | | | | | |
| p191 | | | | | | up | | | | | | | | | | | | | | | | NA | | | | | | | | | | | | | | | | | |  | | | | | | | | | | | | | | | | | | | | | |  | | | cell division protein FtsH-like protein | | | | | | | | | | 77 | | | | | | | | | | | 072367_3859_2372_2p | | | | | | | | | | | | | | | |
| p357 | | | up | | | | | | | | | | | | | | | | | | | | | | | 5.40/147942 | | | | | | | | | | | | | | 74 | | | | | | | | | | | | | | | | gi|242066450 | | | | | | | | | Peptidases_S8_Tripeptidyl_Aminopeptidase_II | | | | | | | | | |  | | | | | | | | | | |  | | | | | | | | | | | | | | | |
| p328|p664|p130 | | | up | | | | | | | | | | | | | | | | | | | | | | | 5.48/75235 | | | | | | | | | | | | | | 431 | | | | | | | | | | | | | | | | gi|84468324 | | | | | | | | | putative zinc dependent protease | | | | | | | | | | 254 | | | | | | | | | | | Contig3343_2p_4 | | | | | | | | | | | | | | | |
| p200 | | | | | | up | | | | | | | | | | | | | | | | 5.83/76015 | | | | | | | | | | | | | | | | | | | | | 206 | | | | | | | | | | | | gi|1483215 | | | | | | | | | | chloroplast FtsH protease | | | | | | | | | | 109 | | | | | | | | | | | Contig28749_2r_1 | | | | | | | | | | | | | | | |
| p399 | | | | | | up | | | | | | | | | | | | | | | | 5.80/74312 | | | | | | | | | | | | | | | | | | | | | 317 | | | | | | | | | | | | gi|147809607 | | | | | | | | | | cell division protein | | | | | | | | | | 98 | | | | | | | | | | | 126915_1464_1042_1r_1 | | | | | | | | | | | | | | | |
| p139|p86 | | | | | | up | | | | | | | | | | | | | | | | 6.00/54643 | | | | | | | | | | | | | | | | | | | | | 221 | | | | | | | | | | | | gi|266567  gi|222424629 | | | | | | | | | | Mitochondrial-processing peptidase subunit alpha | | | | | | | | | | 227 | | | | | | | | | | | 234760_0781_2015_2p  Contig36731_2p_1 | | | | | | | | | | | | | | | |
| p442 | |  | | | | | | | | | | | | | | | | | | | | | | | | 5.59/27213 | | | | | | | | | | | | | | 134 | | | | | | | | | | | | | | | | gi|224139394 | | | | | | | | | predicted protein, proteasome [Populus trichocarpa] | | | | | | | | | | 144 | | | | | | | | | | | Contig10396_1p_1 | | | | | | | | | | | | | | | |
| p457 | | | up | | | | | | | | | | | | | | | | | | | | | | | 4.25/15650 | | | | | | | | | | | | | | 102 | | | | | | | | | | | | | | | | gi|239937266 | | | | | | | | | cysteine protease [Lactuca sativa] | | | | | | | | | | 109 | | | | | | | | | | | Contig13910_3r_8 | | | | | | | | | | | | | | | |
| **Detoxificaiton Defence and Stress Response** | | | | | | | | | | | | | | | | | | | | | | | | | | | | | | | | | | | | | | | | | | | | | | | | | | |  | | | | | | | | | | |  | | |  | | | | | | | | | |  | | | | | | | | | | |  | | | | | | | | | | | | | | | |
| p126|p189 | | | | | | | | |  | | | | | | | | | | | | | | | | | NA | | | | | | | |  | | | | | | | | | | | | | | | | | | | | | | | | | | | |  | | | dehydration stress-induced protein | | | | | | | | | | 165 | | | | | | | | | | | Contig31528_2r_1 | | | | | | | | | | | | | | | |
| p573|p107* | | | up | | | | | | | | | | | | | | | | | | | | | | | 9.61/31275 | | | | | | | | | | | | | | | | | 226 | | | | | | | | | | | | | gi|38679339 | | | | | | | | | harpin binding protein 1 [Zea mays] | | | | | | | | | | 126 | | | | | | | | | | | Contig9087_1p | | | | | | | | | | | | | | | |
| p285 | | | | | |  | | | | | | | | | | | | | | | | | | | | 9.01/25296 | | | | | | | | | | | | | | | | | 145 | | | | | | | | | | | | | gi|242049924 | | | | | | | | | Rhodanese Homology Domain (RHOD) [Sorghum bicolor] | | | | | | | | | | 179 | | | | | | | | | | | Contig3308_2p_1 | | | | | | | | | | | | | | | |
| p346 | | | | | | | | |  | | | | | | | | | | | | | | | | | 5.31/13235 | | | | | | | | | | | | | | | | | 183 | | | | | | | | | | | | | gi|45533923 | | | | | | | | | glycine-rich RNA-binding protein RGP-1c[Nicotiana sylvestris] | | | | | | | | | | | | 429 | | | | | | Contig17268_2p_2 | | | | | | | | | | | | | | | | | | |
| p473 | | up | | | | | | | | | | | | | | | | | | | | | | | | | NA | | | | | | |  | | | | | | | | | | | | | | | | | | | | | | | | | | | |  | | | Peroxiredoxin | | | | | | | | | | 99 | | | | | | | | | | | Contig38348_2p_5 | | | | | | | | | | | | | | | |
| p244 | | | up | | | | | | | | | | | | | | | | | | | | | | | 6.67/95677 | | | | | | | | | | | | | | | | | 77 | | | | | | | | | | | | | gi|218196275 | | | | | | | | | ABC_NikE_OppD_transporters | | | | | | | | | |  | | | | | | | | | | |  | | | | | | | | | | | | | | | |
| p349 | | |  | | | | | | | | | | | | | | | | | | | NA | | | | | | | | | | | | | | | | | | | | |  | | | | | | | | | | | | | | | | | | |  | | | ferritin [Conyza canadensis] | | | | | | | | | | 92 | | | | | | | | | | | Contig7767_3p_1 | | | | | | | | | | | | | | | |
| **Others** | | | | | | | | | | | | | | | | | | | | | | | | |  | | | | | | | | | | | | | |  | | | | | | | | | | | |  | | | | | | | | | | |  | | |  | | | | | | | | | |  | | | | | | | | | | |  | | | | | | | | | | | | | | | |
| p32 | | | | | | up | | | | | | | | | | | | | | | | | | | | 5.20/17463 | | | | | | | | | | | | | | | | | | 80 | | | | | | | | | | | | gi|212720942 | | | | | | | | | hypothetical protein LOC100193724[Zea mays] | | | | | | | | | | | | | | | | | | | | |  | | | | | | | | | | | | | | | |
| p397 | | | up | | | | | | | | | | | | | | | | | | | | | | | | 5.04/14713 | | | | | | | | | | | | | | | | 109 | | | | | | | | | | | | | | gi|132270 | | | | | | | | Rubber elongation factor protein | | | | | | | | | |  | | | | | | | | | | |  | | | | | | | | | | | | | | | |
| p411 | | | | | |  | | | | | | | | | | | | | | | | 6.43/28995 | | | | | | | | | | | | | | | | | | | | | | | 83 | | | | | | | | | | gi|255587064 | | | | | | | | | | ef-hand calcium binding protein, putative[Ricinus communis] | | | | | | | | | | 73 | | | | | | | | | | | Contig1107_2p_2 | | | | | | | | | | | | | | | |
| p326 | | | up | | | | | | | | | | | | | | | | | | | | | | | | 8.88/54682 | | | | | | | | | | | | | | | | 215 | | | | | | | | | | | | | | gi|20743 | | | | | | | | pine globulin-1 [Pinus strobus] | | | | | | | | | |  | | | | | | | | | | |  | | | | | | | | | | | | | | | |
| **(Continued)** | | | | | | | | | | | | | | | | | | | | | | | | | | | | | | | | | | | | | | | | | | | | | | | | | | | | | | | | | | | | | | | | | | | | | | | | | | | | | | | | | | | | | | | | | | | | | | | | | | | | | |
| **Spot ID** | | | **Changes*^a^*** | | | | | | | | | | | | | | | | | | | | | | | | **pI/Mr** | | | | | | | | | | | | | | | | | **Best protein score*^b^*** | | | | | | | | | | | | | | | **Homolog_GI** | | | | | **Protein Name** | | | | | | **EST best protein score** | | | | | | | | | | | | | | | | | | | | | | **Best matching EST ID** | | | | | | | | | |
| p45 | | up | | | | | | | | | | | | | | | | | | | | | | | | 5.20/55158 | | | | | | | | | | | | | | | | | | | 79 | | | | | | | | | | | | gi|115459778 | | | | | | | | Os04g0550400 [Oryza sativa (japonica cultivar-group)] | | | | | | | | | |  | | | | | | | | | | |  | | | | | | | | | | | | | | | |
| p124 | |  | | | | | | | | | | | | | | | | | | | | | | | | NA | | | | | | | | | | | | | | | | | | |  | | | | | | | | | | | |  | | | | | | | | F5I10.22 gene product [Arabidopsis thaliana] | | | | | | | | | | 82 | | | | | | | | | | | 087095_0622_0875_2p_7 | | | | | | | | | | | | | | | |
| **Unknown** | |  | | | | | | | | | | | | | | | | | | | | | | | |  | | | | | | | | | | | | | | | | | | |  | | | | | | | | | | | |  | | | | | | | |  | | | | | | | | | |  | | | | | | | | | | |  | | | | | | | | | | | | | | | |
| p366 | | up | | | | | | | | | | | | | | | | | | | | | | | | 10.36/29623 | | | | | | | | | | | | | | | | | | | 78 | | | | | | | | | | | | gi|125573095 | | | | | | | | hypothetical protein OsJ_04535 [Oryza sativa Japonica Group] | | | | | | | | | | | |  | | | | | | | | |  | | | | | | | | | | | | | | | |
| p742 | | up | | | | | | | | | | | | | | | | | | | | | | | | 5.00/158452 | | | | | | | | | | | | | | | | | | | 75 | | | | | | | | | | | | gi|226458260 | | | | | | | | predicted protein [Micromonas pusilla CCMP1545] | | | | | | | | | |  | | | | | | | | | | |  | | | | | | | | | | | | | | | |
| p561 | |  | | | | | | | | | | | | | | | | | | | | | | | | 4.45/53322 | | | | | | | | | | | | | | | | | | | 75 | | | | | | | | | | | | gi|115488812 | | | | | | | | Os12g0517000 [Oryza sativa (japonica cultivar-group)] | | | | | | | | | |  | | | | | | | | | | |  | | | | | | | | | | | | | | | |
| p68 | | up | | | | | | | | | | | | | | | | | | | | | | | | 6.10/7078 | | | | | | | | | | | | | | | | | | | 74 | | | | | | | | | | | | gi|10716604 | | | | | | | | hypothetical protein [Oryza sativa] | | | | | | | | | |  | | | | | | | | | | |  | | | | | | | | | | | | | | | |
| p81 | |  | | | | | | | | | | | | | | | | | | | | | | | | 5.10/53043 | | | | | | | | | | | | | | | | | | | 74 | | | | | | | | | | | | gi|6143899 | | | | | | | | unknown protein[Arabidopsis thaliana] | | | | | | | | | |  | | | | | | | | | | |  | | | | | | | | | | | | | | | |
| p475 | | up | | | | | | | | | | | | | | | | | | | | | | | | 11.44/53043 | | | | | | | | | | | | | | | | | | | 73 | | | | | | | | | | | | gi|22324448 | | | | | | | | hypothetical protein [Oryza sativa Japonica Group] | | | | | | | | | |  | | | | | | | | | | |  | | | | | | | | | | | | | | | |
| p303 | | up | | | | | | | | | | | | | | | | | | | | | | | | 9.66/15246 | | | | | | | | | | | | | | | | | | | 72 | | | | | | | | | | | | gi|168030864 | | | | | | | | predicted protein [Physcomitrella patens subsp. patens] | | | | | | | | | |  | | | | | | | | | | |  | | | | | | | | | | | | | | | |
| p253 | | up | | | | | | | | | | | | | | | | | | | | | | | | 6.20/148525 | | | | | | | | | | | | | | | | | | | 71 | | | | | | | | | | | | gi|240254562 | | | | | | | | unknown protein [Arabidopsis thaliana] | | | | | | | | | |  | | | | | | | | | | |  | | | | | | | | | | | | | | | |
| p78 | |  | | | | | | | | | | | | | | | | | | | | | | | | 9.47/35948 | | | | | | | | | | | | | | | | | | | 74 | | | | | | | | | | | | gi|168010293 | | | | | | | | predicted protein [Physcomitrella patens subsp. patens] | | | | | | | | | |  | | | | | | | | | | |  | | | | | | | | | | | | | | | |
| p159 | | up | | | | | | | | | | | | | | | | | | | | | | | | 5.32/17505 | | | | | | | | | | | | | | | | | | | 87 | | | | | | | | | | | | gi|255637280 | | | | | | | | unknown [Glycine max] | | | | | | | | | |  | | | | | | | | | | |  | | | | | | | | | | | | | | | |
| **ESTs with no homology to known genes** | | | | | | | | | | | | | | | | | | | | | | | | | | | | | | | | | | | | | | | | | | | | | | | | | | |  | | | | | | | | | | |  | | |  | | | | | | | | | |  | | | | | | | | | | |  | | | | | | | | | | | | | | | |
| p119 | up | | | | | | | | | | | | | | | | | | | | | | | | | | NA | | | | | | | | | | | | | | | | |  | | | | | | | | | | | | | |  | | | | | | |  | | | | | | | | | | 100 | | | | | | | | | | | Contig31239_3p_1 | | | | | | | | | | | | | | | |
| p536 |  | | | | | | | | | | | | | | | | | | | | | | | | | | NA | | | | | | | | | | | | | | | | |  | | | | | | | | | | | | | |  | | | | | | |  | | | | | | | | | | 160 | | | | | | | | | | | Contig40779_2p_1 | | | | | | | | | | | | | | | |
| p523 |  | | | | | | | | | | | | | | | | | | | | | | | | | | NA | | | | | | | | | | | | | | | | |  | | | | | | | | | | | | | |  | | | | | | |  | | | | | | | | | | 116 | | | | | | | | | | | Contig20688_1r_1 | | | | | | | | | | | | | | | |
| p343 | up | | | | | | | | | | | | | | | | | | | | | | | | | | NA | | | | | | | | | | | | | | | | |  | | | | | | | | | | | | | |  | | | | | | |  | | | | | | | | | | 141 | | | | | | | | | | | Contig16569_1p_11 | | | | | | | | | | | | | | | |
| p304 |  | | | | | | | | | | | | | | | | | | | | | | | | | | NA | | | | | | | | | | | | | | | | |  | | | | | | | | | | | | | |  | | | | | | |  | | | | | | | | | | 134 | | | | | | | | | | | Contig17501_2r_2 | | | | | | | | | | | | | | | |
| p389 |  | | | | | | | | | | | | | | | | | | | | | | | | | | NA | | | | | | | | | | | | | | | | |  | | | | | | | | | | | | | |  | | | | | | |  | | | | | | | | | | 86 | | | | | | | | | | | 237117_1464_0273_3p | | | | | | | | | | | | | | | |
| p152 |  | | | | | | | | | | | | | | | | | | | | | | | | | | NA | | | | | | | | | | | | | | | | |  | | | | | | | | | | | | | |  | | | | | | |  | | | | | | | | | | 78 | | | | | | | | | | | 277247_0225_3091_1r | | | | | | | | | | | | | | | |
| p447 | up | | | | | | | | | | | | | | | | | | | | | | | | | | NA | | | | | | | | | | | | | | | | |  | | | | | | | | | | | | | |  | | | | | | |  | | | | | | | | | | 74 | | | | | | | | | | | 233564_2556_0114_3p_4 | | | | | | | | | | | | | | | |
| p393 | up | | | | | | | | | | | | | | | | | | | | | | | | | | NA | | | | | | | | | | | | | | | | |  | | | | | | | | | | | | | |  | | | | | | |  | | | | | | | | | | 73 | | | | | | | | | | | 201199_3918_1922_3r_1 | | | | | | | | | | | | | | | |
| **Spot ID** | | | | | | | | | | | | | | **Changes*^a^*** | | | | | | | | | | | | | | **pI/Mr** | | | | | | | | | | | | | | | | **Best protein score*^b^*** | | | | | | | | | | | | | | | | **Homolog_GI** | | | | | **Protein Name** | | | | | | | | | | **EST best protein score** | | | | | | | | | | | **Best matching EST ID** | | | | | | | | | | | | | | | |
| p131 | | | | | | | | |  | | | | | | | | | | | | | | | | | | | | NA | | | | | | | | | | | | | |  | | | | | | | | | | | | | | |  | | | | | | |  | | | | | | | | | | 136 | | | | | | | | | | | Contig3481_1p_2 | | | | | | | | | | | | | | | |

**^△^**It also includes the proteins p87|p298|p675|p604|p144|p42|p316|p257|p288. *****The best protein result in NCBI database is different from that in EST database; the protein with higher protein score was chosen. *^a^*up-reglated proteins in trichomes; *^b^* the highest score was chosen even if multiple protein spots were attributed to the same proteins. *^c^*calculated pI/Mr values are different from experiment ones; reasons are given in the discussion. *^d^*pI/Mr values were not available in EST database for the proteins predicted in the EST database are short. Note: oxygen-evolving enhancer protein, ribulose-1,5- bisphosphate carboxylase, chloroplast light-harvesting chlorophyll a/b-binding protein, photosystem II reaction center psbP protein, oxygen evolving protein of photosystem, Manganese-stabilizing protein, photosystem I subunit VII, photosynthetic electron transfer-like protein, Ribulose bisphosphate carboxylase/ oxygenase activase, ribulose-1,5-bisphosphate carboxylase/oxygenase large/small subunit were omitted from the table

LITERATURE CITED

1. Teoh, K.H.; Polichuk, D.R.; Reed, D.W.; Nowak, G.; Covello, P.S. *Artemisia annua* L. (Asteraceae) trichome-specific cDNAs reveal CYP71AV1, a cytochrome P450 with a key role in the biosynthesis of the antimalarial sesquiterpene lactone artemisinin. *FEBS Lett*. **2006**, *580*, 1411-1416.
